# Specific serum and CSF microRNA profiles distinguish sporadic behavioural variant of frontotemporal dementia compared with Alzheimer patients and cognitively healthy controls

**DOI:** 10.1371/journal.pone.0197329

**Published:** 2018-05-10

**Authors:** Johannes Denk, Felix Oberhauser, Johannes Kornhuber, Jens Wiltfang, Klaus Fassbender, Matthias L. Schroeter, Alexander E. Volk, Janine Diehl-Schmid, Johannes Prudlo, Adrian Danek, Bernhard Landwehrmeyer, Martin Lauer, Markus Otto, Holger Jahn, for the FTLDc study group

**Affiliations:** 1 Department of Psychiatry and Psychotherapy, University Medical Center Hamburg-Eppendorf, Hamburg, Germany; 2 Department of Psychiatry and Psychotherapy, Friedrich-Alexander-University of Erlangen-Nuremberg, Erlangen, Germany; 3 Department of Psychiatry and Psychotherapy, University Medical Center Goettingen, Goettingen, Germany; 4 Department of Neurology, Saarland University, Homburg, Germany; 5 Clinic for Cognitive Neurology, University Clinic Leipzig and Max Planck Institute for Human Cognitive and Brain Sciences, Leipzig, Germany; 6 Institute of Human Genetics, University Medical Center Hamburg-Eppendorf, Hamburg, Germany; 7 Department of Psychiatry, Technical University of Munich, Munich, Germany; 8 Department of Neurology, University of Rostock, Rostock, Germany; 9 Department of Neurology, Ludwig-Maximilians-University, Munich, Germany; 10 Department of Neurology, University of Ulm, Ulm, Germany; 11 Department of Psychiatry and Psychotherapy, University of Wuerzburg, Wuerzburg, Germany; 12 AMEOS Klinikum, Heiligenhafen, Heiligenhafen, Germany; University of Tennessee Health Science Center, UNITED STATES

## Abstract

Information on circulating miRNAs in frontotemporal lobar degeneration is very limited and conflicting results have complicated an interpretation in Alzheimer’s disease thus far. In the present study we I) collected samples from multiple clinical centers across Germany, II) defined 3 homogenous patient groups with high sample sizes (bvFTD n = 48, AD n = 48 and cognitively healthy controls n = 44), III) compared expression levels in both CSF and serum samples and IV) detected a limited set of miRNAs by using a MIQE compliant protocol based on SYBR-green miRCURY assays that have proven reliable to generate reproducible results. We included several quality controls that identified and reduced technical variation to increase the reliability of our data. We showed that the expression levels of circulating miRNAs measured in CSF did not correlate with levels in serum. Using cluster analysis we found expression pattern in serum that, in part, reflects the genomic organization and affiliation to a specific miRNA family and that were specifically altered in bvFTD, AD, and control groups. Applying factor analysis we identified a 3-factor model characterized by a miRNA signature that explained 80% of the variance classifying healthy controls with 97%, bvFTD with 77% and AD with 72% accuracy. MANOVA confirmed signals like miR-320a and miR-26b-5p at BH corrected significance that contributed most to discriminate bvFTD cases with 96% sensitivity and 90% specificity and AD cases with 89% sensitivity and specificity compared to healthy controls, respectively. Correlation analysis revealed that miRNAs from the 3-factor model also correlated with levels of protein biomarker amyloid-beta_1-42_ and phosphorylated neurofilament heavy chain, indicating their potential role in the monitoring of progressive neuronal degeneration. Our data show that miRNAs can be reproducibly measured in serum and CSF without pre-amplification and that serum includes higher expressed signals that demonstrate an overall better ability to classify bvFTD, AD and healthy controls compared to signals detected in CSF.

## Introduction

The role of microRNAs (miRNAs) in neurodegenerative disorders has gained growing interest in the field [1]. This is due to matured technologies, which now enable the reliable detection of miRNAs in body fluids [2]. MiRNAs are small non-coding RNA that selectively bind different messengerRNAs (mRNA) to downregulate its translation into proteins. These ~22 nucleotides long molecules control fundamental biological processes such as neurogenesis, immune responses and aging and are critical to cellular expression homeostasis [3]. In addition, the transport of miRNAs in extracellular vesicles such as exosomes secreted by neurons and glia plays a key role in intercellular communication and neuroinflammation [4]. The fact that miRNAs are released as circulating miRNAs into the bloodstream not only enables detection, but deregulated signals may reflect neurodegenerative conditions that occur in AD [1, 5]. Thus, miRNAs are easily accessible in minimally invasive body fluids such as serum and cerebrospinal fluid (CSF) [6] and are also known to remain largely stable towards freeze-thawing cycles and RNAse activity [7, 8]. Detection by RT-qPCR is accurate and inexpensive and potentially easier to integrate into clinical routine than assays detecting proteins. A deeper investigation of the role of miRNAs may also foster our understanding of neurodegenerative diseases and lead to new therapeutic approaches.

In contrast to AD, frontotemporal lobar degeneration (FTLD) is a neurodegenerative disorder for which information on circulating miRNAs is very limited. Our knowledge on the miRNome is still scarce. FTLD is characterized by a progressive degeneration of the frontal and anterior temporal lobes causing pathological changes in behaviour and language. About 10–30% of FTLD cases have a known genetic predisposition. Mutations in genes like *C9orf72*, *GRN* (progranulin), *MAPT* (microtubule associated protein tau) or *TBK-1* (encoding TANK-binding kinase 1) can cause FTLD [9–12]. Autosomal dominant inherited cases often occur clustered in families [13]. The large majority of sporadic FTLD cases is, however, of unknown etiology, albeit genetic alterations are to be expected there too [14]. The behavioural variant (bvFTD) accounts for more than half of the cases [15] and is histopathologically described by distinct inclusion bodies either comprised of Tau (FTLD-TAU) [16] or ubiquitinated TDP-43 (FTLD-TDP) [17]. Despite considerable efforts, in-vivo biomarkers are not yet available for FTLD.

We hypothesize that miRNAs in serum and CSF may serve as biomarkers differentiating patients with bvFTD, AD and cognitively healthy controls. Hence, we designed a study following guidelines such as the “minimum information for publication of quantitative real-time PCR experiments” (MIQE) [18] by using SYBR-green based LNA assays (Exiqon A/S), which have proven reliable to measure miRNAs in body fluids compared to other technologies. We profiled two customized miRNA panels each including n = 96 comparable assays in 48 bvFTD, 48 AD and 44 control CSF and serum samples to identify possible biomarker pattern.

## Methods

### Ethics statement

Collection and analysis of samples were approved by the local Ethics Committees of Departments of Neurology and Psychiatry participating in the German FTLD Consortium, a quality-controlled, monitored, multicenter initiative (Ulm approval number 20/10) [19]. All participants of the registry gave their written informed consent for all investigations and their data were fully anonymized prior to any entries in the database. The data we worked with were fully anonymized. All investigations were carried out according to international Good Laboratory Practice (GLP) and Good Clinical Practice (GCP) standard.

### Patient data

Our study population included n = 140 CSF and n = 131 serum samples of bvFTD (48 CSF / 48 serum), AD (48 CSF / 47 serum) and cognitively healthy control cases (HC) (44 CSF / 38 serum). For each case a pair of CSF and serum material was available except for n = 9 serum samples. All samples were provided by the German consortium for frontotemporal lobar degeneration (FTLDc), which were collected from 10 academic centers across Germany [19]. All bvFTD patients met standard diagnostic criteria according to Rascovsky et al. (2011) [20]. Alzheimer’s disease (AD) was diagnosed according to criteria from the National Institute of Neurological and Communicative Diseases and Stroke (NINCDS)–Alzheimer’s Disease and Related Disorders Association [21]. Participants were assessed with extensive diagnostic tools including physical and neurological examination, clinical laboratory testing and analysis of CSF, genetic screening of *C9orf72*, *GRN*, *MAPT* for pathogenic mutations. CSF was obtained by lumbar puncture in a sitting position according to standard procedures [22]. 4 ml CSF was collected into a polypropylene test tube for routine diagnosis as well as for further studies. CSF was free of blood contaminations and tested for hemoglobin. CSF was centrifuged (1600 g, RT, 10 min) and frozen within 30 min after the puncture at -80°C until use. Blood was drawn in 7.5 ml S-Monovette Serum Gel Z tubes (Sarstedt, Germany), incubated for 10 min at RT, centrifuged (2000 g, 4°C, 10 min) and frozen within 30–40 min after blood sampling at -80°C until use. The CSF and serum samples were at no time thawed/refrozen.

### Genetic analyses

DNA was available from 61 participants. Screening of *C9orf72* for pathogenic repeat expansion was performed by amplicon length analysis and repeat-primed PCR was conducted in 61 samples. In case of a suspected expansion, southern blot analysis was performed for confirmation (Akimoto et al., 2014) [23]. In samples from participants with a familial history of a neurodegenerative disease, all exons and flanking intronic regions in *MAPT* and *GRN* were screened by Sanger sequencing (details available upon request). The sequencing results were compared to reference sequences (GenBank entry NM_005910, NM_002087) using the SEQUENCE pilot software (JSI medical systems).

### Laboratory markers

The concentration of albumin, IgG, IgA and IgM in CSF and sera was determined as described earlier (Jesse et al., 2011) [24]. Samples were analysed in the neurochemical lab at the department of Neurology in Ulm. Commercially available ELISA kits were used for Neurofilament light chain (NfL) (IBL, Hamburg, Germany), phosphorylated Neurofilament heavy chain (pNfH) (Biovendor, Heidelberg, Germany), Progranulin (Biovendor, Heidelberg, Germany), Tau, pTau and amyloid-beta_1-42_ (Fujirebio, Hannover, Germany) according to the manufacturer’s instructions.

### RNA isolation

Total RNA, including miRNA, was purified from 250 μl cell-free CSF and 200 μl serum samples using the miRNeasy Serum/Plasma Kit (Qiagen, P/N 217184). The protocol was applied according to the manufacturer’s recommendations with the following modifications. QIAzol Lysis Reagent mixture containing 1.25 μg/ml MS2 bacteriophage RNA (Roche Applied Science) and 1 μl RNA spike-in template mixture containing synthetic UniSp2, UniSp4, and UniSp5 (Exiqon, P/N 203203) was added to all samples. The total RNA was not treated with DNase and eluted with 22 μl RNAse-free water and stored at -80°C until use.

### RT-qPCR

Total RNA was reverse transcribed using the Universal cDNA synthesis kit II, (Exiqon, P/N 203301). Briefly, cDNA was synthesized using 8 μl of total RNA isolated from CSF or serum including 1 μl of a spike-in mix containing synthetic UniSp6 and cel-39-3p (Exiqon, P/N 203203) using a poly-T primer with a 3’ degenerate anchor and a 5’ universal tag in a 20 μl reaction. Reverse transcription was performed at 42°C for 60 min and 95°C for 5 min on a qTower 2.2 (Jena Analytik). 10 μl cDNA was diluted 50x with RNase-free water and added to 500 μl 2x PCR Master mix according to the protocol for the miRCURY LNA Universal RT microRNA PCR System (Exiqon A/S). 10 μl of each sample was assayed on a custom Pick-&-Mix microRNA PCR panel containing pre-spotted LNA primers (S1 Table). Both the CSF and the serum panel each consisted of n = 96 abundant and highly expressed miRNAs. N = 76 miRNAs were identical on both panels, but n = 20 different. All miRNAs were selected based on a preliminary pilot study [1] and a comparable study by Mooney et al. [25], that both used OpenArray technology and pre-amplification. A no-template control (NTC) of RNase-free water was co-purified and profiled like the samples to measure background. Amplification was performed on a qTower 2.2 (Jena Analytik) using the following thermal cycling protocol: 95°C for 10 min, 45 amplification cycles at 95°C for 10 s and at 60°C for 60 s (fluorescent reading), followed by a melting curve analysis.

### Pre-processing, quality control and normalisation

We adapted our RT-qPCR protocol to the MIQE guidelines in order to provide sufficient experimental details to increase the reliability of our data (S2 Table) [18]. Raw unprocessed fluorescence values were exported from the qTower 2.2 and analysed with the “qpcR” package [26]. Cq values were calculated by using the second derivative maximum after fitting a 5-parameter sigmoidal model to the fluorescence data [27]. In addition, qPCR runs with kinetic outliers were identified and removed [28]. Furthermore, all signals showing < 80% valid data after applying the following QC criteria were excluded from analysis: (1) Signals with Cq ≥ 37 and dCq ≤ 3 (Ct_NTC_−Ct_miR-x_) were considered background, (2) all assays were inspected for distinct melting curves and (3) serum samples with a dCq ≥ 7 (Cq_miR-23a_ −_miR-451_) were considered at high risk of hemolysis [29].

NormFinder and GeNorm were used to identify potential reference miRNAs using GenEx [30, 31]. CSF samples were normalised to miR-101-3p, miR-320a and miR-99a-5p. Serum samples were normalised to let-7i-5p, miR-23a-3p, miR-23b-3p and miR-30e-5p. Relative expression was calculated as follows: *dCt* = *Ct* (*Ct_mean RefmiR_* − *Ct_miR_*) and *ddCt* = *mean dCt_AD or bvFTD_* − *mean dCt_HC_*. Higher values indicate higher expression. Only signals with a ddCt ≥ |0.58|, which corresponds to a fold change of ≥ |1.5|, were considered as differentially regulated.

### Absolute quantification

Synthetic oligos that match mature miRNA sequences (S3 Table) were ordered from IDT (Integrated DNA Technologies) to prepare an initial pool of oligos each with a concentration of 33 nM. Standard curves were generated by preparing 4-fold serial dilutions (n = 7) each at a volume of 80 μl containing 0.8 μg/μl MS2 carrier RNA (Roche) following RT-qPCR as described earlier. Standards were measured as cDNA duplicates and PCR triplicates and assayed on a custom Pick-&-Mix microRNA PCR Panel containing n = 96 pre-spotted LNA primers (S3 Table). Background was measured with a no template control including water. Standard curves were obtained by fitting a regression line to the samples with known concentrations.

### Statistics

Two-tailed unpaired Mann-Whitney t-tests at a significance level of 5% were used to determine statistical differences between two groups and chi square tests for dichotomous variables to examine demographic group differences. One-way MANOVA was applied to identify statistical differences between more than two groups, respectively. *P* < 0.05 (two sided) was considered as statistically significant. The false discovery rate was controlled by using the Benjamini–Hochberg procedure when conducting multiple comparisons. Correlations were determined using Pearson r for normally distributed data or the Spearman test when data were not normally distributed at a 5% significance level. To evaluate diagnostic accuracy we used receiver operating curves and discriminant analysis. Unsupervised hierarchical clustering and differential correlations were calculated by using the DiffCorr v0.4.1 package for R [32].

Exploratory Factor Analysis (EFA) permits examination of how unmeasured latent variables (factors) summarize patterns of correlations found in the measured relationships between miRNA expression levels. The following criteria for the factorability of a correlation were used: 1) correlation of all variables with at least 0.3 with at least one other variable, 2) the Kaiser-Meyer-Olkin (KMO) measure of sampling adequacy for each variable must be ≥ 7.5 and Bartlett’s test of sphericity should be significant, 3) the diagonals of the anti-image correlation should be above 0.5, 4) the communalities above 0.3 and 5) only miRNAs based on factor loadings ≥ |0.5| were considered significant in contributing to the respective factor. A scree plot assisted of how many factors should be extracted. The eigenvalues indicate how much of the variance is explained by each factor. Calculation and graphics were done using XLStat (v19.4), GraphPad (v7.03) and SPSS (v24) software and the R 3.4.2 statistical programming language (R Development Core 2008).

## Results

### Characteristics of study population

As summarized in **Table 1**, no significant difference was observed in the distribution of age and gender. The bvFTD group had higher MMSE scores compared to the AD group (p<0.05). Classical protein biomarkers Tau and pTau were increased in CSF in the AD patients compared to the bvFTD and control cases (p<0.0001). Conversely, amyloid-beta_1-42_ levels were decreased in both the AD and bvFTD group compared to the controls (p<0.0001). We also measured levels of the neurofilament light and the phosphorylated heavy chain but detected large variation in CSF. NfL levels were observed significantly higher (p<0.05) in bvFTD cases and pNFH levels were higher (p<0.05) in AD cases compared to controls. Since we were primarily interested in the sporadic, non-genetic bvFTD, we examined our samples for mutations in the genes *C9orf72*, *GRN* and *MAPT*. A total of 41 of the 48 bvFTD and 20 of the 48 AD cases were tested negative for the most prominent gene *C9orf72*. Further, no mutations in the genes *MAPT* and *GRN* were identified in the tested AD (n = 11) and bvFTD (n = 11) cases. To address possible center-effects, we compared the baseline miRNA expression data of the study sites (S1 Fig).

**Table 1:** Demographic and descriptive parameters for study population.

Characteristic^1^ / samples	HC (CSF n = 44) (serum n = 38)^2^	bvFTD (CSF n = 48) (serum n = 48)	AD (CSF n = 48) (serum n = 47)^2^	p-value (ANOVA / x^2^)		
				AD vs HC	AD vs bvFTD	bvFTD vs HC
**Age (years)**	64 ± 11.3	65 ± 9.2	65 ± 9.3	ns	ns	ns
**Sex (male/female)**	20/24	30/18	22/26	ns	ns	ns
**MMSE**	nm	24 ± 4	21 ± 5.3	na	< .05	na
**Tau [pg/ml]**	317.6 ± 118.8	433.4 ± 414.7	738 ± 288.9	< .0001	< .0001	ns
**pTau [pg/ml]**	35.1 ± 6.7	59.4 ± 37.2	96 ± 38.2	< .0001	< .0001	< .05
**Aβ**_**1–42**_ **[pg/ml]**	1031.5 ± 272.2	872.1 ± 338	513 ± 160.6	< .0001	< .0001	< .05
**NfL [pg/ml]**	1449.1 ± 940.2	2706.4 ± 1816.2	2157.6 ± 1026.4	ns	ns	< .05
**pNfH [pg/ml]**	307.6 ± 151.8	464.56 ± 253.85	540.33 ± 244.96	< .05	ns	ns
**Tested / not tested for mutation in *C9orf72*, *MAPT*, *GRN* (n)**	na	41 / 7 11 / 37 11 / 37	20 / 28 11 / 37 11 / 37	na	na	na
**Tested negative for mutation in *C9orf72*/*MAPT*/*GRN***	nm	41 11 11	20 11 11	na	na	na

^1^ Data represent mean ± SD analysed by 1-way ANOVA (age and CSF biomarker) and t-test (MMSE). Gender ratio was analysed by χ2.

^2^ Reduced number of serum samples did not lead to a significant change in demographical data. AD = Alzheimer’s disease, bvFTD = behavioural variant frontotemporal dementia, HC = healthy controls, na = not applicable, nm = not measured.

### Quality controls indicate low technical variation and consistent detection of miRNA levels in CSF and serum

With a mean Cq of 33.61 ± 2.2, the expression of circulating miRNAs in CSF was > 26-fold lower compared to serum with a mean Cq of 28.89 ± 3.66. This was also reflected by the increased number of positive signals detected for each miRNA in our serum data. In total, 77 miRNAs displayed ≥ 80% positive signals per miRNA in serum while this only applied to 55 miRNAs in CSF. RNA isolation was monitored by using a subset of synthetic miRNAs that simulate high (UniSp2), medium (UniSp4) and low (UniSp5) expressed signals to measure and compare extraction efficiency [29]. cDNA synthesis was controlled by UniSp6 and cel-miR-39. Our results demonstrate constant extraction efficiency across all samples with acceptable intra-assay variation as well as constant efficiency of the reverse transcription step with no signs of inhibition (S2A Fig). However, two serum samples were removed due to suspicious expression of spikes. We also checked the degree of hemolysis in our serum samples. The obtained data were not affected by erythrocyte miRNA contamination. Only a minority of serum samples indicated a possible but no high risk of hemolysis (S2B Fig). Using Normfinder and GeNorm, we were able to identify a subset of miRNAs for CSF and serum that considerably reduced technical variation compared to un-normalized data (S2C and S2D Fig). Furthermore, standard curves based on synthetic miRNA oligos were generated for a subset of assays to assess PCR efficiency and to estimate copy numbers. For the selected set of miRNA assays, PCR efficiencies were within an acceptable range of E = 0.95–0.98, which demonstrated low variation and good assay performance (S3 Fig). We also measured CSF and serum samples isolated at different days to test the inter-assay variance of our protocol. SYBR-green miRNA assays displayed consistent expression with low inter-assay variance for serum (R^2^ = 0.99, F(1,70) = 11078, p < .0001) and CSF (R^2^ = 0.97, F(1,74) = 2215, p < .0001) showing a trend towards increasing day-day variation at higher expression levels as seen in the CSF data (S4 Fig).

### Expression levels of circulating miRNAs in serum do not correlate with levels in CSF

Following quality control and the removal of low expressed signals, we were able to compare 34 of the 76 identical CSF and serum miRNA assays in 131 samples. Taking into account all subjects, an average correlation of r = 0.077 (p = ns) indicated no association of miRNA expression levels in serum compared to expression levels in CSF. If we considered expression levels of individual miRNAs, we observed that some of these correlated significantly between serum and CSF, however, only with weak (r<0.3) associations. This finding applied to all three subgroups. Taking into account the mean values ​​of the respective subgroups, the control (r = 0.051, p = ns), bvFTD (r = 0.077, p = ns) and AD (r = 0.057, p = ns) group displayed no significant associations. However, expression levels of a few miRNAs showed significant correlations of moderate extent in the respective subgroups. This applied to both miR-19b-3p (r = 0.37, p = 0.023) and miR-25-3p (r = 0.45, p = 0.011) in the control group. Significant positive correlations of miRNA expression levels in serum with expression levels in CSF were also found for miR-143-3p (r = 0.34, p = 0.023), miR-29b-3p (r = 0.33, p = 0.025) and miR-29c-3p (r = 0.44, p = 0.002) in the bvFTD group. Interestingly, miR-24-3p showed a significant negative correlation, r = -0.33, p = 0.026. In the AD group, serum expression levels of let-7f-5p (r = 0.32, p = 0.032), miR-100 (r = 0.36, p = 0.017) and miR-143-3p (r = 0.32, p = 0.047) correlated positive with levels in CSF. In turn, we observed a significant negative correlation of miR-197-3p (r = -0.42, p = 0.005) and miR-30a-5p (r = -0.38, p = 0.012).

### Cluster analysis of serum expression levels reveals miRNA families and genomic clusters altered in bvFTD and AD samples

**Fig 1:**
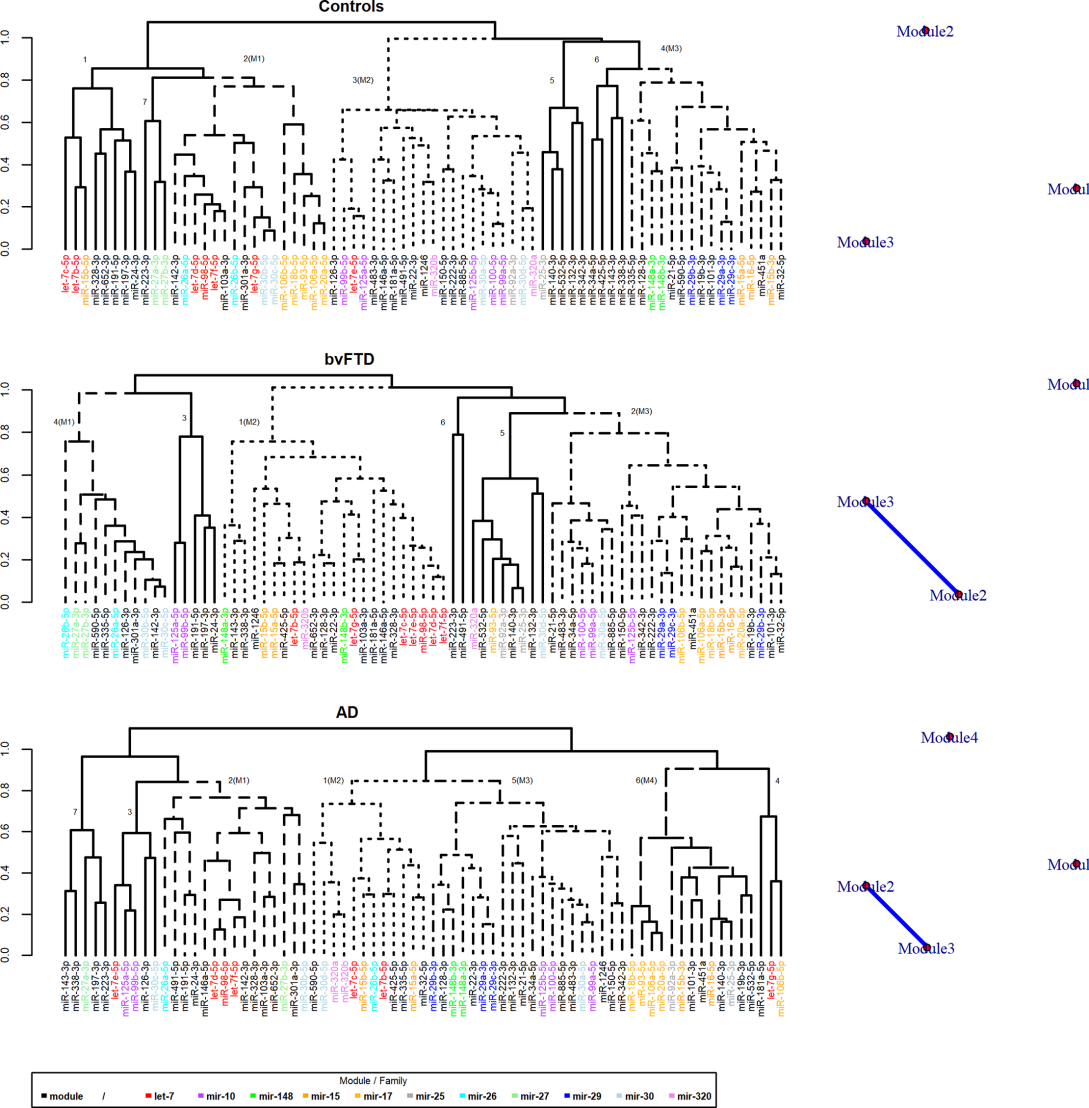
Unsupervised hierarchal clustering of miRNA levels measured in serum of healthy control, bvFTD and AD samples. Using the DiffCorr package, the genes were grouped according to their expression patterns in each subtypes (cognitively healthy controls, bvFTD and AD) using the cluster.molecule function. We used (1 − correlation coefficient) as a distance measure (the cutoff value was a coefficient of 0.6) based on the cutree function. We then visualized the module network using the get.eigen.molecule and get.eigen.molecule.graph functions. MiRNAs that share similar seed sequences (miRNA families) are coloured. MiRNAs that are co-transcribed as a polycistronic unit (http://www.mirbase.org/, < 10 kb) are listed in S5 Table. AD = Alzheimer’s disease, bvFTD = behavioural variant frontotemporal dementia, M = Module.

As a first approach to understand the interaction of miRNAs, hierarchical clustering was applied to the serum dataset containing 131 samples measured for the expression of 96 miRNAs. To reduce the noise, we excluded miRNAs with low expression values (detailed in Material and Methods). Using average linkage bottom up clustering a total of 7 clusters in the control, 6 clusters in the bvFTD and 7 clusters in the AD group were detected (**Fig 1**). These clusters each contain 2–24 miRNAs (**Fig 1**). Notably, many cluster contained miRNAs with similar sequences (miRNA families) as well as miRNAs of a polycistronic unit that share a common promotor (referred to as genomic cluster, http://www.mirbase.org/, <10 kb). Genomic clusters containing only members of a particular family (homo-clusters) as well as clusters containing miRNAs with different seed sequences (hetero-clusters) were present. A good example for co-expression related to similar sequences is Cluster 7 in the control group that contains hsa-miR-27a and hsa-miR-27b or hsa-miR-148a and hsa-miR-148b in Cluster 4. In contrast, the genomic cluster let-7e/miR-99b/125a in Cluster 3 is an example of a hetero-cluster. In addition, we identified at least three Modules (dotted lines) in each condition. Modules are clusters of highly interconnected miRNAs that are characterized by dense interactions. Interestingly, the modules differed not only in terms of number and composition of miRNAs between the conditions but Modules 2 and 3 were also identified as densely connected groups in the bvFTD and AD group, which was not observed in the control group. As expected, we identified several pair-wise differential correlations in each condition with a trend of increased correlations in the bvFTD group. This was supported by the fact that some families such as mir-17 or let-7 or clusters such as miR-29b/29a and miR-29b/29c appeared more intermixed in the bvFTD condition. S4 Table shows the top 20 significantly differential co-expressed miRNAs (FDR<0.05) in bvFTD cases compared to cognitively healthy controls.

### Factor analysis reveals miRNA structure in serum, which allows the classification of bvFTD, AD and control cases

The variety of differential correlations in the individual conditions made an interpretation difficult. This led us to consider all samples simultaneously using factor analysis to reduce the number of manifest variables to a few hypothetical variables that could be associated with the diagnoses of our patient samples. The goal of factor analysis is to detect a small set of factors that elucidate as much of the variance of the output variables as possible.

**Fig 2:**
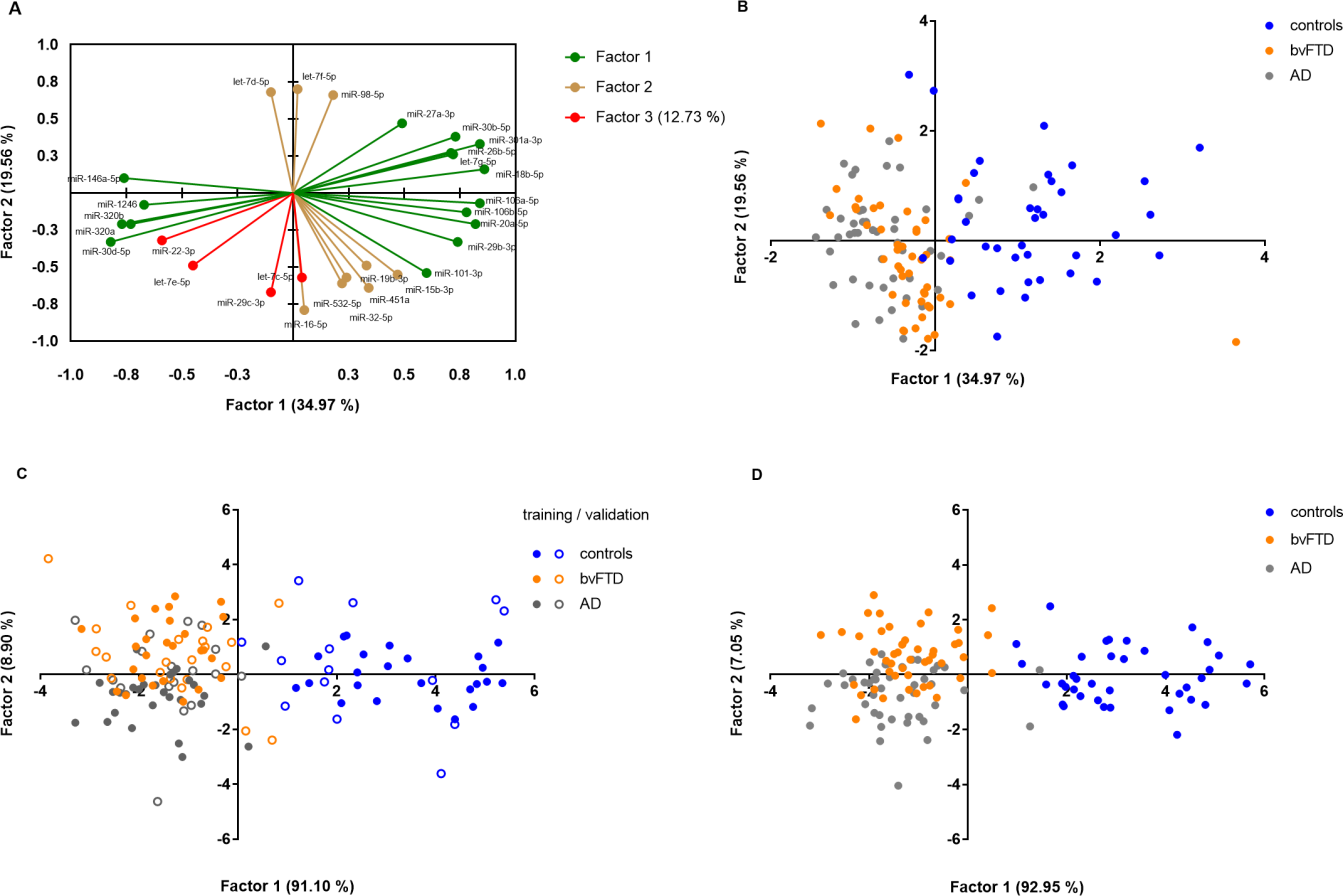
Factor and discriminant analysis of miRNA signals detected in serum. We have used factor and discriminant analysis to identify the variables that can best characterize and classify the bvFTD, AD and healthy control samples in our serum cohort. The following results are shown: (A) factor model of n = 29 serum miRNAs that load on the Factors 1–3 with factor loadings ≥ |0.5|, (B) observation plot showing coordinates of the observations resulting from the two-factor model (Factors 1–2), and result of the discriminant analysis using a re-factoring 2-factor model based on the n = 29 miRNAs from the original 3-factor model (**Fig 2A**) using (C) a training (n = 78) and validation (n = 53) set and (D) all cases (n = 131).

The factorability of 73 miRNAs in serum was initially examined. Applying several well-recognised criteria [33–35], 29 miRNAs were tested, which led to an acceptable subject-to-item ratio of ~ 1:5. A total of 44 variables were eliminated because they either failed to meet the minimum criteria or did not contribute to a simple factor structure by primary factor loadings of <|0.5|. All items in this analysis had primary loadings over .5. Some of the variables showed cross-loadings, but most showed strong primary loadings and were therefore not excluded. Looking at the scree plot of the serum data, eigenvalues showed that in each case 34.97%, 19.56%, 12.73% and accordingly a total of 67.26% of the variation were explained by the first three factors (S5 Fig). The fourth factor also had an eigenvalue > 1, but explained only a small part of the total variability with 4.72%. As a result, the three-factor solution was preferred to the four-factor model as depicted in **Fig 2**. **Fig 2A** shows how the initial miRNAs are correlated with the three factors. We could observe that Factor 1 (green) positively correlated with hsa-let-7g-5p, -miR-101-3p, -106a-5p, -106b-5p, -18b-5p, -20a-5p, -26b-5p, -29b-3p, -301a-3p, -30b-5p and -27a-3p and negatively correlated with hsa-miR-1246, -146a-5p, -30d-5p, -miR-320a and -320b. In contrast. Factor 2 (red) positively correlated with hsa-let-7d-5p, -let-7f-5p and -miR-98-5p and negatively correlated with hsa-miR-15b-3p, -16-5p, -32-5p, -451a, -532-5p and -19b-3p whereas Factor 3 (brown) negatively correlated with hsa-let-7c-5p, -let-7e-5p, -miR-22-3p and miR-29c-3p. The factor loading matrix for this final solution is presented in S6 Table. Interestingly, the use of the coordinates of the observations of the two-factor model already resulted in a significant discrimination of cognitively healthy controls from the bvFTD and AD group (**Fig 2B**). More interestingly, the structure also visually indicated a trend towards a grouping of AD and bvFTD cases, however, with a certain number of false positive signals, respectively (**Fig 2B**). To improve the differentiation of the samples, the miRNAs of the third factor, which also contributed with 12.73% to the total variance, were taken into account in a subsequent discriminant analysis as shown in **Fig 2C**. After the data was split into a training- (n = 78) and validation (n = 53) set, 84.62% and respectively 60% of the total cases were successfully classified based on the analysis (**Fig 2C**). If the individual subgroups were considered, 100% of the controls, 70% of the bvFTD and 86% of the AD cases of the training set were correctly classified (**Fig 2C**). On the other hand, the validation set showed that 71% of controls, 67% of bvFTD and 44% of AD cases were correctly assigned (**Fig 2C**). Considering the entire data set (n = 131 serum samples), the controls were correctly predicted with 97%, the bvFTD cases with 77% and the AD cases with 72% accuracy (**Fig 2D**).

Looking at the CSF data, n = 15 miRNAs were suitable for factor analysis. This resulted in an acceptable subject-to-item ratio of ~ 1:9. The scree plot indicated that the first three factors had eigenvalues >1, which explained 26.33%, 24.25% and 9.29% of the variance, respectively (S5 Fig). Since we did not observe a trend towards a valuable separation of our samples using the CSF miRNA factor model, we further concentrated on our serum data.

### Expression analysis identifies significantly de-regulated miRNAs between bvFTD, AD and control samples

Next, we tested whether the miRNAs of the 3-factor model as well as other miRNAs in our CSF and serum data displayed significantly different expression levels and to what extent these signals contributed to the classification of our samples by calculating corresponding AUC values. Using MANOVA, we identified a number of miRNAs, all of which were differentially expressed over a ddCt of |0.58| (S7 Table). Except for miR-30d-5p and miR-101-3p, all signals of Factor 1 were significantly de-regulated. In contrast, only miR-22-3p of Factor 3 was found as differentially expressed but none of the signals from Factor 2.

**Fig 3:**
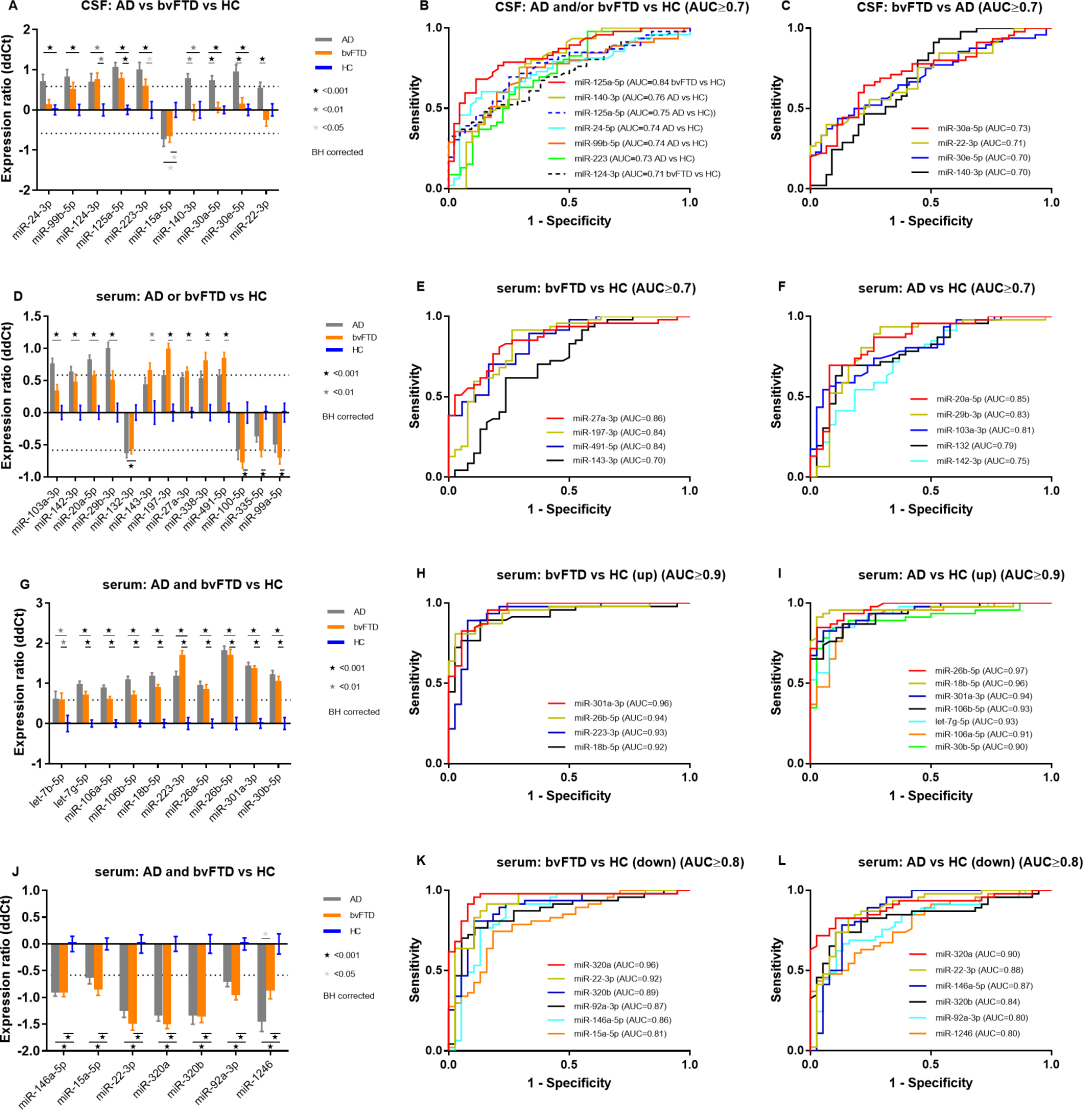
Differentially expressed miRNAs in bvFTD, AD and cognitively healthy control cases detected in CSF and serum. Expression levels of n = 96 circulating miRNAs were measured in CSF (n = 140) and serum (n = 131) samples from bvFTD (n = 48/48) and AD patients (n = 48/47) as well as healthy controls (n = 44/38) and compared using MANOVA and ROC curves. Displayed are signals with significantly different expression levels after multiple comparisons detected in (A) CSF: group comparisons of AD, bvFTD and healthy controls with (B-C) corresponding ROC curves and (D) serum: group comparisons of AD, bvFTD and healthy controls (up- and downregulated miRNAs) with (E-F) corresponding ROC curves, (G) serum: group comparisons of AD, bvFTD and healthy controls (only upregulated miRNAs) with (H-I) corresponding ROC curves and (J) serum: group comparisons of AD, bvFTD and healthy controls (only downregulated miRNAs) with (K-L) corresponding ROC curves. Expression ratio: *ddCt* = *mean dCt_AD or bvFTD_* − *mean dCt_HC_*. Dotted lines indicate ddCt cut-off of |0.58|. Error bars indicate mean ± SEM. BH = Benjamini-Hochberg.

In our CSF data, we identified a total of 10 miRNAs with significantly different expression levels (**Fig 3A**). The best classifier was miR-125a-5p that discriminated bvFTD cases with 72% sensitivity and 81% specificity as well as AD cases with 74% sensitivity and 82% specificity from our controls (**Fig 3B**). In contrast, we also observed miRNAs with significantly different expression levels between AD and bvFTD patients (**Fig 3A**), which showed more moderate classification performance. With an AUC of 0.73 (95% CI, 0.623–0.832), miR-30a-5p yielded the best classification by separating bvFTD from AD cases with 78% sensitivity and 68% specificity (**Fig 3C**).

In our serum data, a total of n = 31 miRNAs were identified with significantly different expression levels between our subgroups. Two basic expression patterns could be observed. One was characterized by signals that were differentially regulated in the bvFTD or AD group compared to the controls (**Fig 3D**). Here, bvFTD cases separated best from controls with 77% sensitivity and 72% specificity by miR-27a with an AUC of 0.86 (95% CI, 0.775–0.935) (**Fig 3E**). In contrast, miR-20a-5p demonstrated an AUC of 0.85 (95% CI, 0.768–0.938) and the highest specificity of 92%, whereas miR-29b-3p displayed an AUC of 0.83 (95% CI, 0.738–0.931) by separating AD cases from controls with 93% sensitivity (**Fig 3F**). In the second expression pattern, miRNAs were either significantly up- or downregulated both in bvFTD and AD patients compared to controls (**Fig 3G and 3J**). For example, miR-301a-3p demonstrated an AUC of 0.96 (95% CI, 0.918–0.996) and classified bvFTD cases with high sensitivity (96%) and specificity (84%), whereas miR-26b-5p showed and AUC of 0.97 (95% CI, 0.940–0.999) and classified AD cases with 89% sensitivity and specificity with respect to our control group (**Fig 3H and 3I**). Accordingly, miR-320a was the best classifier against controls observed as collectively down regulated in bvFTD and AD. ROC analysis resulted in an AUC of 0.96 (95% CI, 0.909–1.003), which classified bvFTD cases with 96% sensitivity and 90% specificity and an AUC of 0.90 (95% CI, 0.835–0.969) to predict AD cases with 83% sensitivity and 90% specificity (**Fig 3K and 3I**).

We also examined the respective groups on a gender-specific basis. As expected, most signals showed the same differentially expressed levels in both men and women. Interestingly, in our serum data, we identified signals with increased classification performance to classify bvFTD from AD in the male but not in the female cohort compared to the original analysis that considered both males and females. Signals miR-103a-3p (AUC = 0.80), miR-106a-5p (AUC = 0.80) and miR-1246 (AUC = 0.85) demonstrated increased sensitivities (70–77%) and specificities (75–85%) to classify bvFTD from AD cases. This trend was also overserved in our CSF data but less significant in terms of classification performance.

### MiRNAs from factor model in serum correlate with levels of amyloid-beta_1-42_ and neurofilaments light chain detected in CSF

**Fig 4:**
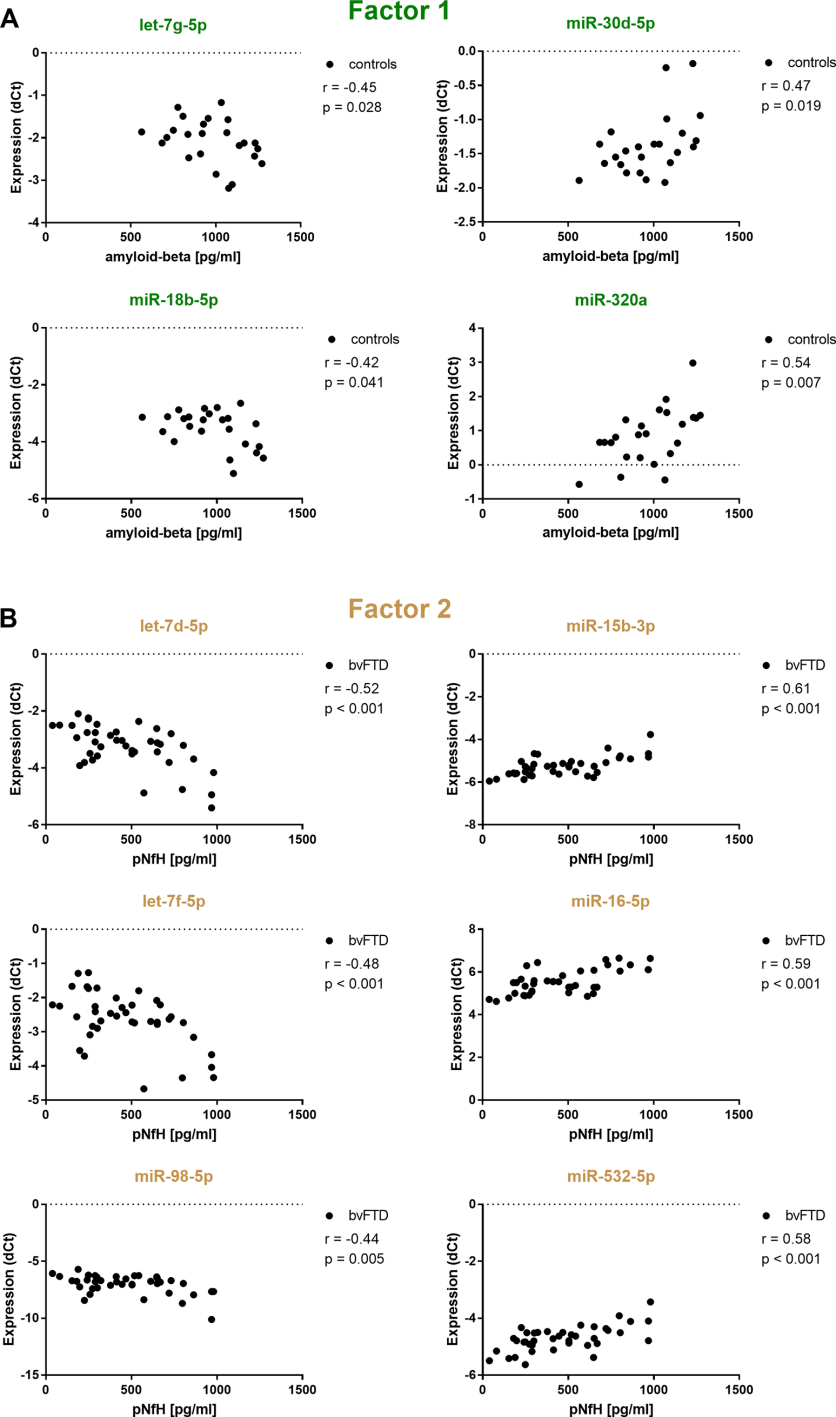
Correlations of miRNA expression levels in serum with CSF protein biomarkers. Depicted are normalized expression levels *dCt* = *Ct* (*Ct_mean RefmiR_* − *Ct_miR_*) of (A) miRNAs from the original 3-factor model that correlated with Factor 1 vs CSF levels of amylod-beta_1-42_ in the control group and (B) miRNAs from the original 3-factor model that correlated with Factor 2 vs CSF levels of pNfH in the bvFTD group. pNfH = phosphorylated neurofilament heavy chain.

As described previously, we were unable to observe a global relationship between miRNA expression levels in serum and CSF. Instead, only a weak trend towards associations of single signals was observed. However, we were also interested in whether the expression levels of miRNAs correlated with those of classical protein biomarkers in the CSF. As a result, we found a large number of significant correlations of our serum miRNAs with these biomarkers. The most interesting finding was that individual miRNAs that correlated with Factor 1 predominantly correlated with amyloid-beta_1-42_ (**Fig 4A**) whereas miRNAs that correlated with Factor 2 primarily correlated with pNfH (**Fig 4B**). For example, miR-320a (r = 0.54, p = 0.028) showed a significantly positive correlation, whereas miR-18b-5p (r = -0.42, p = 0.042) displayed a significantly negative correlation with CSF amyloid-beta_1-42_ levels. Both signals also positively correlated with Factor 1 (**Fig 2A**). Another example are let-7d (r = -0.52, p<0.001), let-7f (r = -0.48, p<0.001) and miR-98-5p (r = -0.44, p = 0.005), all of which show significantly negative correlations with CSF pNFH levels (**Fig 4B**). These signals were also found to positively correlate with Factor 2 as identified by factor analysis (**Fig 2A**).

## Discussion

In the present study we I) collected samples from multiple clinical centers across Germany, II) defined 3 homogenous patient groups with increased sample sizes (bvFTD n = 48, AD n = 48 and cognitively healthy controls n = 44), III) compared expression levels in both CSF and serum samples and IV) focused on a limited set of miRNAs. Many studies use RT-qPCR to search for circulating miRNA biomarkers but do not consider the MIQE guidelines, do not control for hemolysis in serum or plasma samples, or use an inappropriate normalization method. We included appropriate quality control procedures that identified and reduced known (pre-) analytical sources of variation (S1–S4 Figs) and adapted our protocol to the MIQE guidelines (S2 Table) to increase the experimental transparency and reliability of our data.

One major finding was that we did not observe a strong association of miRNA expression levels throughout the cohort or subgroups between our CSF and serum samples. This is consistent with results from a comparable study by Wang et al. and Freischmidt et al., who also observed a poor association of miRNA levels in CSF and serum [36, 37]. Wang et al., however, showed an increased correlation of CSF miRNA levels with miRNAs from the Choroid Plexus [37]. In our case single miRNAs such as miR-29b and miR-29c indicated a small trend for a correlation (R^2^ = 10–20%) between CSF and serum. However, these miRNAs are known to control fibrinogen production, which is a basic pathway both in the brain and the periphery [38]. In summary, the results point to autonomous networks that may independently respond to neurodegenerative processes and show little similarity in cognitively healthy controls. However, one has to keep in mind that low CSF levels point to low input into CSF and that levels might dilute out during circulation and after blood-brain-barrier transport. This might be another reason why potential changes in CSF cannot necessarily be seen in serum.

The main goal was to identify miRNA signatures to classify our bvFTD, AD and cognitively healthy control cases using multivariate statistics. To increase reliability, we focused on the serum data, which contained higher quality signals compared to the CSF data. One approach towards this aim was to use co-expression analysis. The first step included unsupervised cluster analysis to analyse possible interactions within each subgroup. An interesting observation was that the cluster analysis, which based on cell-free serum expression levels, grouped many signals according to their affiliation with a polycistronic unit (e.g. miR-106a/-b cluster) or a particular miRNA family (e.g. mir-17, let-7, mir-15 or mir-320 family). These (co-)expression patterns are known to occur within tissue cells [39, 40], different blood cell types [41] or whole blood [42]. The result therefore suggested that the profile of circulating miRNAs in serum appears to be more similar to the cell than expected but also point to a more unspecific release into the circulation. Interestingly, Leidinger and colleagues identified similar miRNA clusters and families as specifically enriched in blood cell populations positive for different CD markers, which supports this idea [41]. However, the extent to which members of a miRNA family (independent transcription units) displayed similar expression patterns in serum was unexpected because the mechanisms responsible for interchromosomally-coordinated co-expression are not yet fully understood and are the subject of current research [43]. In the second step we calculated differential correlations to better compare the individual profiles between our subgroups. However, the increased number of differential correlations made the interpretation difficult and prompted us to use factor analysis, which considered samples of all subgroups. The initially 73 serum miRNAs were reduced by ~40% to 29 signals, which in turn resulted in a three-factor miRNA model.

Factor 1 correlated with miR-106b-5p and miR-20a-5p, which in turn belong to the mir-106b~25 and mir-17 cluster. Both were found as significantly upregulated in bvFTD and AD, which is in line with results from Cheng et al, who investigated and validated miRNA levels isolated from serum exosomes using RNA seq [44]. In addition, both signals demonstrated a good to excellent diagnostic accuracy to distinguish AD cases from controls. Interestingly, these miRNAs were also shown to regulate APP *in vitro* and in neuronal cell lines [45]. In addition, we identified a differential correlation pattern for the mir-30b/30d cluster. We observed that miR-30b-5p was positively and miR-30d-5p negatively correlated with Factor 1 and we further confirmed significantly upregulated expression levels in bvFTD and AD compared to our controls. Notably, miR-30b-5p was found to correlate with amyloid plaque density by a study of Burgos et al. [5]. In addition, we found miR-30d-5p (R^2^ = 22%) to positively correlate with CSF amyloid-beta_1-42_ levels in our cognitively healthy controls. This also applied to miR-320a (R^2^ = 30%), which is predicted to downregulate APP [46], whereas its family member miR-320b was found as a possible regulator of human-specific neural development [47]. We could also confirm both signals as significantly down-regulated in bvFTD and AD compared to our cognitively healthy controls. Overall, miR-320a and miR-320b showed a good to excellent diagnostic accuracy to correctly separate bvFTD and AD cases from controls, with miR-30b-5p ranking only AD cases correctly.

Factor 2 correlated positively with the let-7f/7d and let-7f/mir-98 clusters, whose members belong to the let-7 family, but we could not observe significantly different expression levels. However, let-7d and miR-98 were found as significantly down-regulated in AD compared to controls by Burgos et al. [5]. Interestingly, miR-98-5p was also found to act as a target for AD by regulating the production of beta-amyloid through modulating SNX6 Expression [48]. Another study showed, that inhibition of miR-98 in N2a/APP cells up-regulated the IGF-1 protein level and suppressed Aβ production [49]. Interestingly, we found all members of the let-7f/7d (R^2^ = 23–27%) and let-7f/mir-98 (R^2^ = 19%) cluster to negatively correlate with CSF pNfH levels in the bvFTD group. This also applied to miR-532 (R^2^ = 34%) and the mir-15 family members’ miR-15b-3p (R^2^ = 37%) and miR-16-5p (R^2^ = 37%) that negatively correlated with Factor 2. miR-15b-3p displayed significantly upregulated expression in AD and miR-16-5p significantly downregulated expression levels in bvFTD and AD compared to controls, however, each below a ddCt of .58. In addition, both miRNAs correlated significantly positively with CSF pNfH levels in the bvFTD group. Neurofilaments are major proteins of neurons and are particularly concentrated in axons and detection in CSF provides information about the degree of axonal injury [50] and was found a biomarker for genetic frontotemporal dementia [51, 52]. Notably, Burgos et al. identified miR-16-5p to negatively correlate with Braak stage [5], which supports the idea of miR-16-5p as a potential marker of neuronal injury. Furthermore, the miR-15 family has also been shown to modulate Tau phosphorylation through ERK1 leading to neuronal death in Neuro2a cells and primary cortical neurones [53]. However, we did not observe significant correlations with CSF Tau or pTau levels in our groups.

Factor 3 contained no more than 2 members of a specific miRNA family. With the exception of the miR-29 family. In this case, miR-29c-3p correlated negatively with Factor 3, with the family member miR-29b-3p positively correlating with Factor 1. In contrast, miR-29a-3p did not correlate with any of the three factors but was found to negatively correlate with CSF Tau (r = -0.56) and pTau (r = -69) levels (data not shown). However, we only found expression levels of miR-29b-3p as significantly upregulated in AD compared to our controls. Interestingly, Hebert et al. identified the mir-29a/b-1 as significantly decreased in AD brain as well as its regulation of amyloid-beta levels by upregulated levels of BACE1 [54]. miRNAs correlating with Factor 2 and Factor 3 showed an overall lower diagnostic potential compared to those correlated with Factor 1. Except miR-29b-3p (AUC = 0.83) for the classification of AD cases.

In summary, all signals from the three-factor model explained in total > 67% of the variance. We therefore calculated a subsequent discriminant analysis to evaluate the performance of this model to classify our subgroups. As expected from the structure, the miRNA signature was able to identify cognitively healthy controls with 97% accuracy. The result outperforms other blood-based assays such as the detection of amyloid levels [55] and performs at least as well [56–58], if not better [59–61] compared to miRNAs in blood that have been identified in other studies. More interestingly, the same signature was able to classify bvFTD cases with 77% and AD cases with 72% accuracy and can at least in part compete with results from traditional protein based tests in CSF [62]. This is mostly due to the fact that the majority of miRNAs that were either up- or downregulated in AD compared to our cognitively healthy controls displayed a similar expression pattern in bvFTD patients. We believe that these rather unspecific signals are mainly due to the underlying neurodegeneration observed in AD and bvFTD. There were basically no signals on our panel that showed an opposing expression except for those that have been identified in our gender specific analysis as mentioned earlier. However, this should be the focus of further studies as specific signals would improve diagnosis by helping to exclude other dementias.

Concerning our CSF data, we identified individual miRNAs that showed significantly different expression levels and displayed diagnostic potential. For example miR-125a-5p that discriminated cognitively healthy controls from AD with good (AUC = 0.84) or miR-30a-5p that classified bvFTD from AD cases with moderate (AUC = 0.73) accuracy. This is in line with Cogswell, who also identified these signals as significantly upregulated in AD [63]. However, due to quality control we lost some signals in our CSF data and factor analysis did not reveal a miRNA signature with high discriminatory value.

In summary, our data show that our circulating miRNA profile in CSF was not comparable to that in serum and that serum miRNAs were better detectable compared to those in CSF. In addition, circulating miRNAs in serum show a strong tendency to form clusters, either because of their genomic organization or because of homologies in their sequences. Co-expression analysis displayed differently co-expressed miRNAs between our subgroups. However, the identified associations were complex and difficult to interpret. In addition, possible associations of other miRNAs could not be demonstrated, since we did not include related signals on our serum panel. We could also show that multivariate methods such as factor analysis can identify miRNA signatures in serum able to classify bvFTD, AD and control cases with acceptable diagnostic accuracy. Due to their genomic organization and transcriptional expression pattern we therefore think that it is more likely to identify a biomarker consisting of either members of a miRNA cluster or family compared to single miRNAs. Another interesting observation was that particularly miRNAs associated with our factor model also correlated with CSF amyloid-beta_1-42_ and phosphorylated neurofilament heavy chain levels either with our control- or bvFTD group. This suggested that de-regulated miRNAs of a family or cluster may possibly be able to monitor the neurodegenerative progression seen in AD or bvFTD. However, further evidence from cell culture experiments is necessary to better describe the functional associations of these miRNAs. Overall, when analysing miRNA clusters and families, it must be noted that it is based on current understanding and annotation and that this relationship will change over time whenever new miRNA species are identified. However, unsupervised clustering is based on what was actually measured, thus reduces this bias in part and is therefore well suited for the detection of networks. However, one shortcoming towards the identification of miRNA signatures using cluster and factor analysis is the limited number of signals in our study. The (factor-) structures identified by us result from a small part of the miRNome that circulate in serum. However, there are other related (S5 Table) and novel miRNAs that we did not measure but that may further increase complexity. The last miRBase update was 2014. Novel miRNAs have been identified by NGS, but their expression levels have not yet extensively been investigated using qPCR and are not yet available on PCR panels. An updated selection of abundant signals could thus provide novel candidates for miRNA profiling studies. A shortcoming towards our study group was that not all individual were tested for one of the disease causing mutations. However, the probability of carrying one of the tested mutations is generally low and the majority was tested negative so that a few possible mutation carriers should not bias the results. Furthermore, some variation at baseline miRNA expression was observed across the different study sites. We think that (possible) centre effects should affect all miRNAs equally (due to a harmonized and standardised protocol for sampling and extraction) so that variation on a single miRNA basis should not occur. The variation may possibly be the result of a numerically unequal distribution of the patient groups from the different centres. Multi-centre studies should therefore make sure that the proportion of patient groups from the various centres is as equal as possible. Considering the discussed points in follow up studies will greatly contribute to identify other relevant miRNAs to better understand the complex expression pattern overserved in body fluids, and may further improve the classification performance to separate AD and bvFTD cases.

## Supporting information

S1 Fig Baseline expression across all miRNAs between different study sites. A main effect for baseline expression levels (across all miRNAs) between study sites was found in our serum (F(9, 121) = 5.21, p < 0.001) and CSF (F(9, 130) = 4.50, p < 0.001) data. Serum expression levels from the study site in Ulm (M = 29.17, SD = 1.06) were lower compared to München-tu (M = 27.86, SD = 0.61), Homburg (M = 27.74, SD = 0.29), Erlangen (M = 27.9, SD = 0.46) and Hamburg (M = 28.3, SD = 0.93). In contrast, CSF expression levels from the study site in Göttingen (M = 32.42, SD = 1.23) were higher compared to Ulm (M = 33.59, SD = 0.65), München-tu (M = 33.56, SD = 0.49) and Erlangen (M = 33.95, SD = 0.54). However, each difference was below the critical threshold of ddCt < |0.58| except for Göttingen vs. Erlangen (ddCt = 0.62).
(TIF)

S2 Fig Pre-analytical variation of circulating miRNA in CSF and serum samples. a) Box plot (whiskers: 2.5–97.5 percentile) of synthetic miRNAs display low technical variation with acceptable intra-assay variation of UniSp2: Cq_CSF_ 17.59 ± 0.37 and Cq_serum_ 17.49 ± 0.49; UniSp4: Cq_CSF_ 24.92 ± 0.52 and Cq_serum_ 24.42 ± 0.59 and with a trend of increasing variation towards the isolation of lower expressed transcripts like UniSp5: Cq_CSF_ 30.65 ± 0.92 and Cq_serum_ 31.23 ± 0.51. UniSp6: Cq_CSF_ 18.63 ± 0.46 and Cq_serum_ 17.59 ± 0.16 and cel-miR-39 Cq_serum_ 24.07 ± 0.25 were used to monitor the cDNA synthesis reactions and indicated constant RT efficiency with no signs of inhibition. b) The hemolysis plot indicates expression ratios of constant miR-23a and red blood cell sensitive miR-451a to monitor serum samples for signs of cellular contamination or hemolysis. With a mean dCq_miR-23a_ –_miR-451_ = 4.05 ± 1.07, most of the serum samples did not display signs of hemolysis (dCq ≤ 5, yellow line). Only a few signals showed a dCq > 5 but none of the samples appeared at high risk of hemolysis (dCq ≥ 7, red line). c,d) The cumulative distribution plots display different miRNA normalisation strategies applied on the serum and CSF data. Normalization with reference miRNAs identified by NormFinder and GeNorm resembled normalisation to the global mean and considerably reduced technical variation compared to un-normalized data or data normalized to internal standards.
(TIF)

S3 Fig miRNA standard curves for SYBR green miRCURY PCR assays. Plotted are mean Cq values from n = 4 replicate standard curves vs the log2 copy numbers. Standard curves were generated for a subset of assays by using a dilution series of a pool of known input amounts of synthetic miRNA oligonucleotides corresponding to the target sequence of the assay. Red error bars depict mean Cq ± CI. R^2^ = coefficient of determination, E = PCR efficiency ± CI, CI = confidence interval.
(TIF)

S4 Fig Day-to-Day reproducibility. RNA from A) CSF and B) serum isolated with miRNeasy serum Kit on different days was measured on each corresponding 96-well panel. Interplate calibrated Cq values were plotted against each other to assess the degree of inter-assay variability by linear regression. R^2^ = coefficient of determination.
(TIF)

S5 Fig Scree plots of serum and CSF data. The scree plot shows the variance explained by each factor in a factor analysis and is used to assess the optimum number of factors to take into account for further analysis.
(TIF)

S1 Table Raw Cq values of endogenous miRNAs (cut-off Cq < 37) detected in CSF and serum samples. Listed is the FOC (number of positive miRNAs), arithmetic mean of raw Cq values of each subgroup and the standard deviation, the miRBase accession and the miRNA sequence. bvFTD = behavioural variant frontotemporal dementia, Cq = quantification cycle, FOC = frequency of occurrence, HC = cognitively healthy controls, SD = standard deviation.
(XLSX)

S2 Table MIQE checklist. Provided are necessary information recommended by the MIQE guidelines to increase experimental transparency of quantitative real-time PCR experiments.
(XLSX)

S3 Table Sequence information of synthetic oligos used for absolute quantification. (XLSX)

S4 Table Top 20 list of differentially coexpressed miRNAs from the serum dataset. Shown are significantly pair-wise differential correlations (FDR < 0.05) of miRNA expression levels in serum between cognitively healthy controls and bvFTD cases using the comp.2.cc.fdr function from the DiffCorr package. r = Pearson correlation coefficient, lfdr = local false discovery rate.
(XLSX)

S5 Table Table of miRNAs used for hierarchal cluster analysis. Depicted are 1) miRNAs, 2) sequence information, 3) clustered miRNAs on genome (http://www.mirbase.org/, <10 kb), 4) paralogous miRNAs, 5) location on chromosome, 6) miRNA family and 7) correlation with Factor 1–3 from factor analysis. miRNAs in red were not included on our serum panel.
(XLSX)

S6 Table Factor loadings and communalities based on a principal factor analysis for n = 29 miRNAs detected in n = 131 serum samples. Only miRNAs based on factor loadings ≥ |0.5| were considered significant in contributing to the respective factor. F = Factor.
(XLSX)

S7 Table Differentially expressed miRNAs detected in serum and CSF. Listed are miRNAs that displayed significantly different expression levels in either serum or CSF samples between cognitively healthy control, bvFTD and AD cases. Fold change is calculated as *ddCt* = *dCt_CtRef −CtmiRNA_* − *dCt_CtRef −CtmiRNA_* where larger values reflect higher abundance. AD = Alzheimer’s disease, bvFTD = behavioural variant frontotemporal dementia, HC = cognitively healthy controls, p = p-value.
(XLSX)
