# Supplementary figures and images for: Specific serum and CSF microRNA profiles distinguish sporadic behavioural variant of frontotemporal dementia compared with Alzheimer patients and cognitively healthy controls
Source: PLoS One. 2018 May 10;13(5):e0197329. doi: 10.1371/journal.pone.0197329 (PMC5945001; doi:10.1371/journal.pone.0197329)

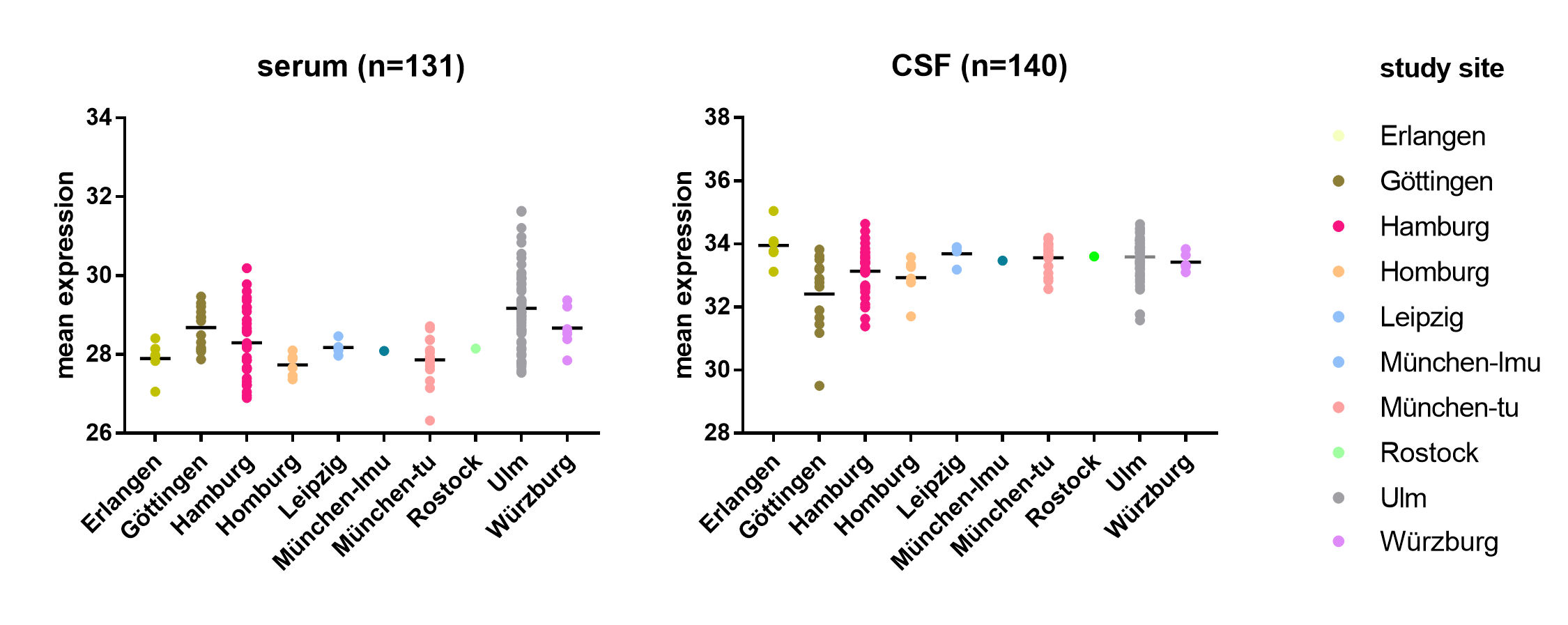

Supplement: S1 Fig — A main effect for baseline expression levels (across all miRNAs) between study sites was found in our serum (F(9, 121) = 5.21, p < 0.001) and CSF (F(9, 130) = 4.50, p < 0.001) data. Serum expression levels from the study site in Ulm (M = 29.17, SD = 1.06) were lower compared to München-tu (M = 27.86, SD = 0.61), Homburg (M = 27.74, SD = 0.29), Erlangen (M = 27.9, SD = 0.46) and Hamburg (M = 28.3, SD = 0.93). In contrast, CSF expression levels from the study site in Göttingen (M = 32.42, SD = 1.23) were higher compared to Ulm (M = 33.59, SD = 0.65), München-tu (M = 33.56, SD = 0.49) and Erlangen (M = 33.95, SD = 0.54). However, each difference was below the critical threshold of ddCt < |0.58| except for Göttingen vs. Erlangen (ddCt = 0.62). (TIF) [file pone.0197329.s001.tif]

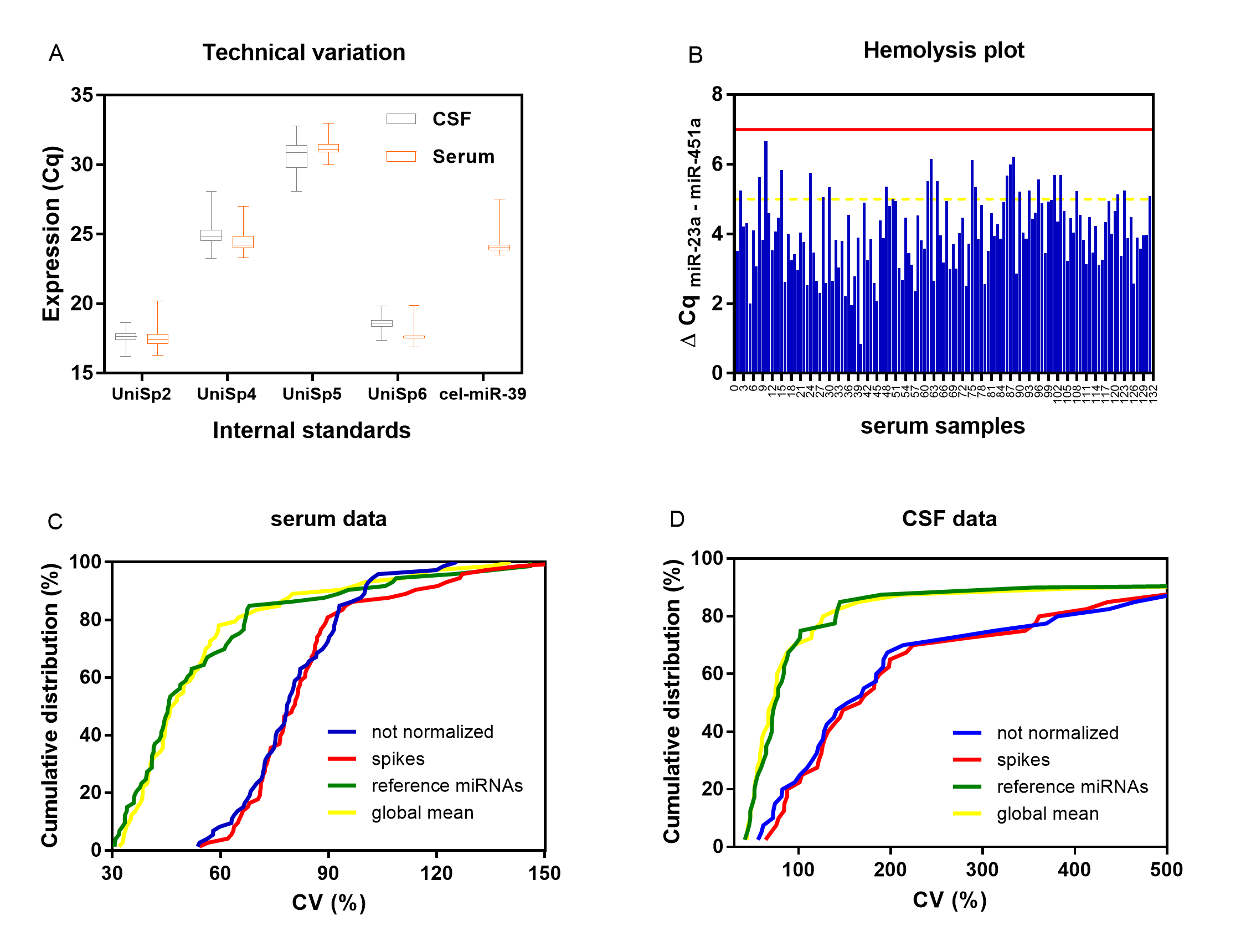

Supplement: S2 Fig — a) Box plot (whiskers: 2.5–97.5 percentile) of synthetic miRNAs display low technical variation with acceptable intra-assay variation of UniSp2: CqCSF 17.59 ± 0.37 and Cqserum 17.49 ± 0.49; UniSp4: CqCSF 24.92 ± 0.52 and Cqserum 24.42 ± 0.59 and with a trend of increasing variation towards the isolation of lower expressed transcripts like UniSp5: CqCSF 30.65 ± 0.92 and Cqserum 31.23 ± 0.51. UniSp6: CqCSF 18.63 ± 0.46 and Cqserum 17.59 ± 0.16 and cel-miR-39 Cqserum 24.07 ± 0.25 were used to monitor the cDNA synthesis reactions and indicated constant RT efficiency with no signs of inhibition. b) The hemolysis plot indicates expression ratios of constant miR-23a and red blood cell sensitive miR-451a to monitor serum samples for signs of cellular contamination or hemolysis. With a mean dCqmiR-23a –miR-451 = 4.05 ± 1.07, most of the serum samples did not display signs of hemolysis (dCq ≤ 5, yellow line). Only a few signals showed a dCq > 5 but none of the samples appeared at high risk of hemolysis (dCq ≥ 7, red line). c,d) The cumulative distribution plots display different miRNA normalisation strategies applied on the serum and CSF data. Normalization with reference miRNAs identified by NormFinder and GeNorm resembled normalisation to the global mean and considerably reduced technical variation compared to un-normalized data or data normalized to internal standards. (TIF) [file pone.0197329.s002.tif]

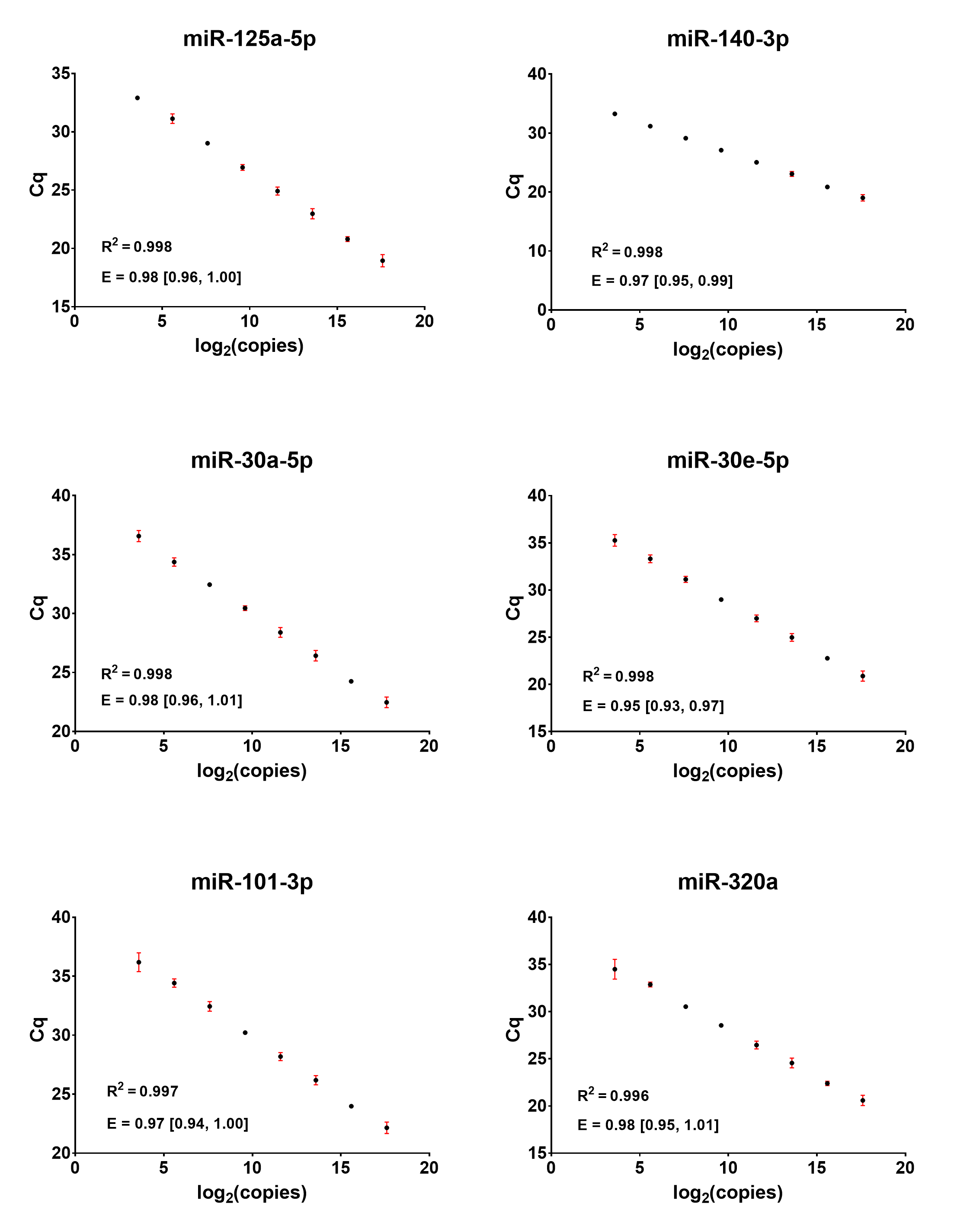

Supplement: S3 Fig — Plotted are mean Cq values from n = 4 replicate standard curves vs the log2 copy numbers. Standard curves were generated for a subset of assays by using a dilution series of a pool of known input amounts of synthetic miRNA oligonucleotides corresponding to the target sequence of the assay. Red error bars depict mean Cq ± CI. R2 = coefficient of determination, E = PCR efficiency ± CI, CI = confidence interval. (TIF) [file pone.0197329.s003.tif]

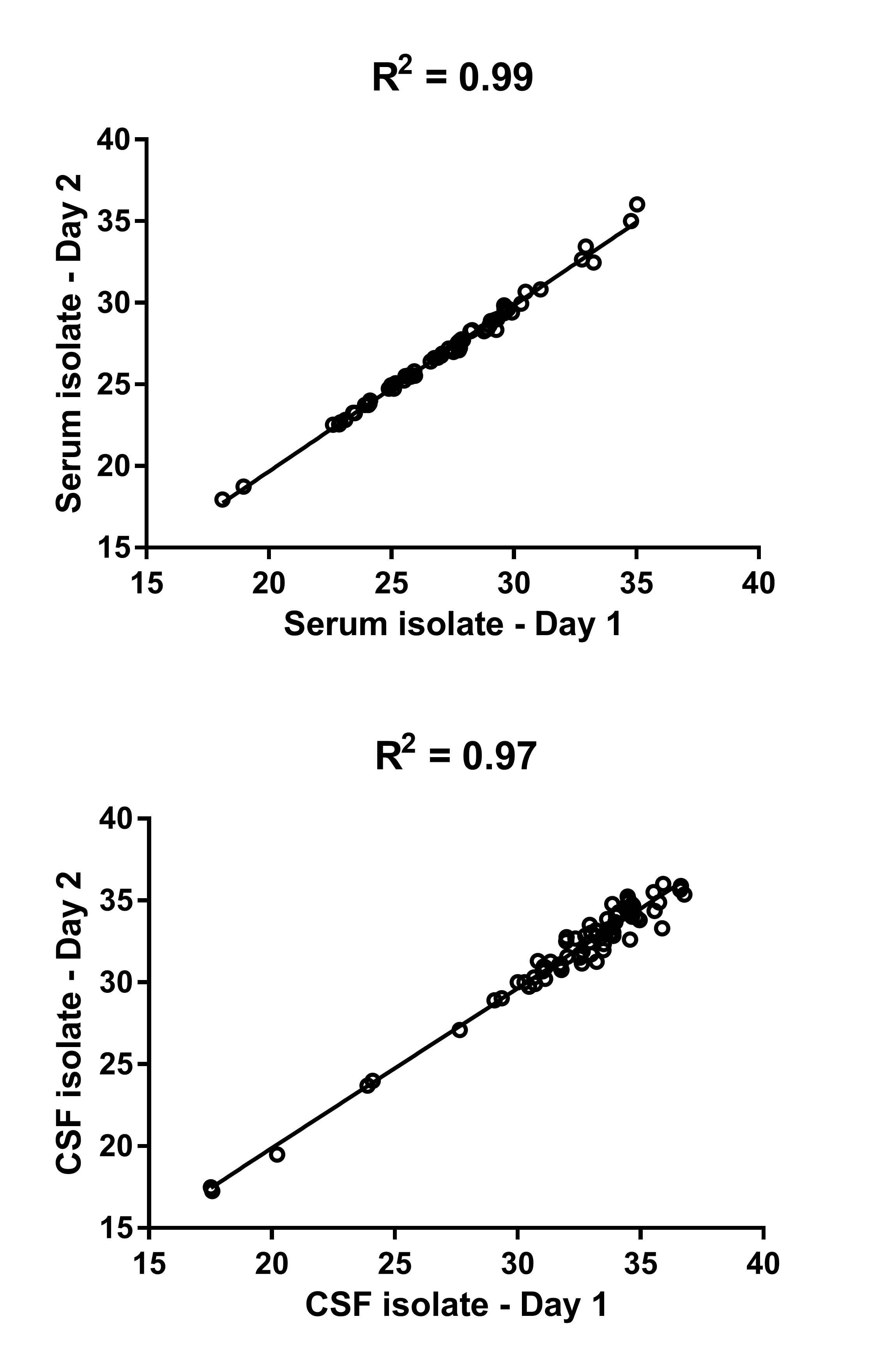

Supplement: S4 Fig — RNA from A) CSF and B) serum isolated with miRNeasy serum Kit on different days was measured on each corresponding 96-well panel. Interplate calibrated Cq values were plotted against each other to assess the degree of inter-assay variability by linear regression. R2 = coefficient of determination. (TIF) [file pone.0197329.s004.tif]

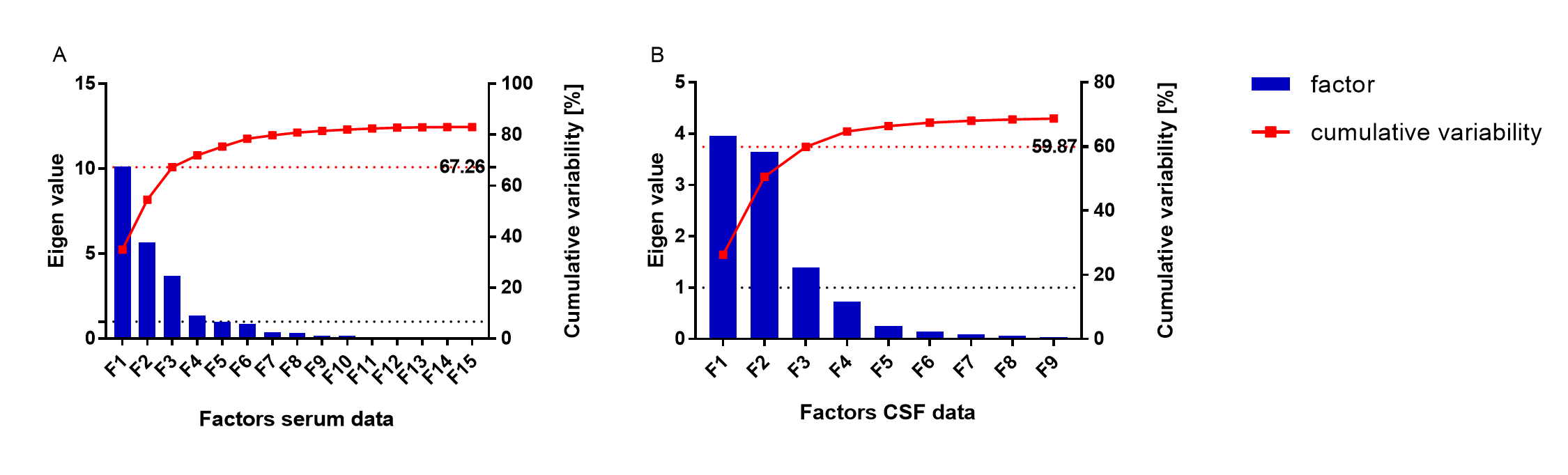

Supplement: S5 Fig — The scree plot shows the variance explained by each factor in a factor analysis and is used to assess the optimum number of factors to take into account for further analysis. (TIF) [file pone.0197329.s005.tif]
